# Scientists May Approach the Bench

**DOI:** 10.1100/tsw.2000.12

**Published:** 2000-11-10

## Abstract

NASA science is lightening up and embracing microgravity research on the International Space Station, reports Nature in this week's lead article. Science's lead story covers a group of litigious tissue donors who are trying to recover control of their genes.

Canavan disease is a fatal genetic illness that strikes as many as 1 in 6400 Ashkenazi Jewish children. Several years ago, reports *Science*, a group of parents called the Canavan Foundation joined forces with scientist Reuben Matalon to track down the deadly gene. The parents group found the tissue donors; Matalon found the gene and developed a screening test in 1993. Then Miami Children's Hospital, Matalon's employer at the time, stepped in. The hospital took out a patent on the gene and started charging a $12.50 royalty fee for the test. The fee forced the Canavan Foundation to stop offering free tests, enraging the parents group and provoking them to sue the hospital for "misappropriation of trade secrets." The hospital isn't backing down, claiming they will "enforce vigorously our intellectual property rights relating to carrier, pregnancy, and patient DNA tests," reports *Science*.

Litigation is also featured in *Science's* number-two story. *Science* reports that the Immune Response Corporation of Carlsbad, California, and researchers at the University of California-San Francisco (UCSF) jointly developed and tested a drug for AIDS treatment called Remune. The UCSF researchers found no evidence of clinical benefit during extensive trials. The company, on the other hand, claims a subset of the data showed that the health of some patients improved. UCSF researchers published their results without the Immune Response data subset; Immune Response has threatened a multi-million dollar suit if they don't retract the article or add the subset.

*Nature's* second slot covers the United States Labor Relations Board ruling that New York University graduate teaching and research assistants qualify as employees. *Science* also covered the story (but placed it farther down) and reports that some professors are predicting that unionization "heralds yet another breakdown in the internal organizational fabric of higher education." How you feel about that apparently depends on who you are. Administrators called the ruling "deplorable" and grad students were "thrilled", reports *Science*.

The two magazines also cover a report that a widely used pesticide causes rats to develop the rigidity and unsteadiness characteristic of Parkinson's Disease in humans. Although the active ingredient of the pesticide rotenonethe is found in hundreds of products from flea and tick powders to tomato sprays, reports *Science*, researchers caution that no link between the rat experiment and Parkinson's disease in humans has yet been established.
